# Search for high-capacity oxygen storage materials by materials informatics†

**DOI:** 10.1039/c9ra09886k

**Published:** 2019-12-17

**Authors:** Nobuko Ohba, Takuro Yokoya, Seiji Kajita, Kensuke Takechi

**Affiliations:** Toyota Central R&D Labs., Inc. Nagakute Aichi 480-1192 Japan e4606@mosk.tytlabs.co.jp; Toyota Motor Corporation Higashi-Fuji Technical Center Susono Shizuoka 410-1193 Japan

## Abstract

Oxygen storage materials (OSMs), such as pyrochlore type CeO_2_–ZrO_2_ (p-CZ), are used as a catalyst support for three-way catalysts in automotive emission control systems. They have oxygen storage capacity (OSC), which is the ability to release and store oxygen reversibly by the fluctuation of cation oxidation states depending on the reducing or oxidizing atmosphere. In this study, we explore high-capacity OSMs by using materials informatics (MI) combining experiments, first-principles calculations, and machine learning (ML). To generate training data for the ML model, the OSC values of 60 metal oxides were measured from the amount of CO_2_ produced under alternating flow gas between oxidizing (O_2_) and reducing (CO) conditions at 973, 773, and 573 K. Descriptors were computed by atomic properties and first-principles calculations on each oxide. The support vector machine regression model was trained to predict the OSC at each temperature. The features describing OSC were automatically selected using grid search to achieve practical cross validation performance. The features related to the stability of the oxygen atoms in the crystal and the crystal structure itself such as cohesive energy are highly correlated with OSC. The present model predicts the OSC of 1300 existing oxides. Based on its high predictive power for OSC and synthesizability, we focused on Cu_3_Nb_2_O_8_. We synthesized this material and experimentally confirmed that Cu_3_Nb_2_O_8_ showed a higher OSC than conventional OSM p-CZ. This MI scheme can significantly accelerate the development of new OSMs.

## Abstract 2

Novel high-capacity oxygen storage material, Cu_3_Nb_2_O_8_, has been discovered by materials informatics.

## Graphical abstract

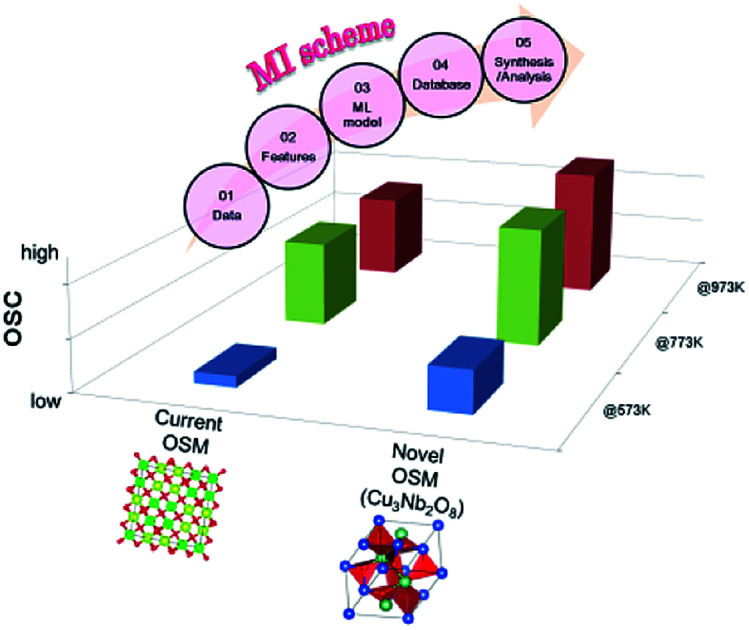

## Introduction

Carbon monoxide (CO), hydrocarbons (HC), and nitrogen oxides (NO_*x*_) emitted from gasoline-powered automobiles are environmentally hazardous substances as they contribute to air pollution and ozone layer depletion. A three-way catalyst system can convert these compounds (CO, HC, and NO_*x*_) into CO_2_, H_2_O, and N_2_ simultaneously. It functions effectively if the weight ratio of air to fuel (air–fuel ratio) in the automobile engine is near the theoretical stoichiometric air–fuel (A/F) ratio. However, actual driving conditions, such as acceleration and deceleration, often make the air-fuel ratio deviate from the ideal stoichiometric value. To control the A/F ratio, an oxygen storage material (OSM)^1,2^ is used as a catalyst support for storing and releasing oxygen. The OSMs incorporate oxygen from an oxidation atmosphere and supply oxygen in a reducing atmosphere; this property is called the oxygen storage capacity (OSC). That is, OSC is defined as the amount of oxygen per OSM weight stored in and released from the OSM on a time scale of seconds or minutes.^3^ OSC is represented as the ability of oxidation-state change of constituent cation in OSM. It is usually measured from the consumption of a reducing agent like H_2_ or CO, or the amount of CO_2_ produced under alternating flow gas between oxidizing(O_2_) and reducing (CO) conditions.^2^ OSMs can regulate the exhaust-gas atmosphere and support the catalysis of the noble metal to convert the compounds properly. For example, CeO_2_ (ref. 4) and CeO_2_–ZrO_2_ (ref. 5) are known for being OSMs currently in use. Because a high OSC broadens the operating range of A/F ratios,^6,7^ novel OSMs that show sufficient OSC, especially at lower temperatures, are desirable for a high conversion efficiency.

**Fig. 1:**
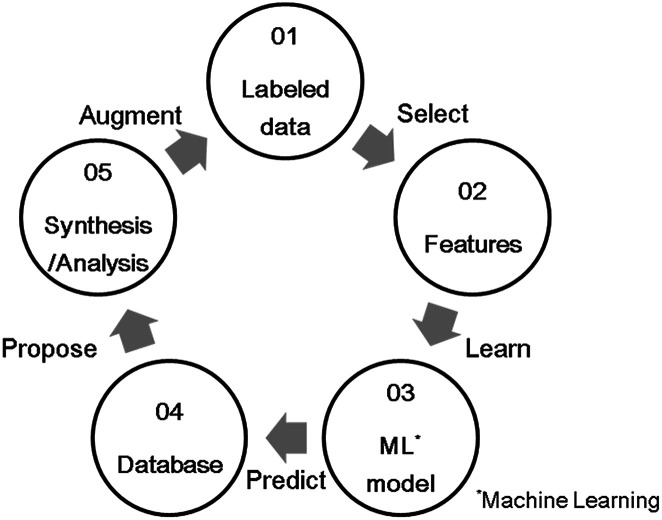
Schematic diagram of a virtual screening scheme by supervised learning.

In recent years, materials informatics (MI) has attracted attention as a method of accelerating the search for new viable materials from a wider range of materials space. MI uses materials data, and constructs a predictive model using machine learning techniques in order to discover new materials that have not yet gained attention.^8,9^ A representative search method in MI is virtual screening based on supervised learning, as shown in Fig. 1. This method has been used in a variety of applications, such as in the search for oxide ion conductors,^10^ organic EL materials,^11^ cathode materials for lithium-ion batteries,^12^ and lithium ion conductors.^13,14^ While there are many researches that have reported to able to accelerate material developments by MI, there are still scarce reports that achieved experimental validations. Indeed, according to a review paper,^15^ only 26 studies have been reported along with experimental validations. One of the difficulties is that experimental data in literature are observed in different conditions. In this study, we utilize a well-defined experiment dataset which were obtained in a unified condition by the same facility, to perform the virtual screening method. The aim of this virtual screening is to discover a novel OSM that indicates a higher OSC than the current state-of-the-art storage materials. The process of virtual screening is outlined in Fig. 1 and this paper is organized along these procedures.

### Training data

**Fig. 2:**
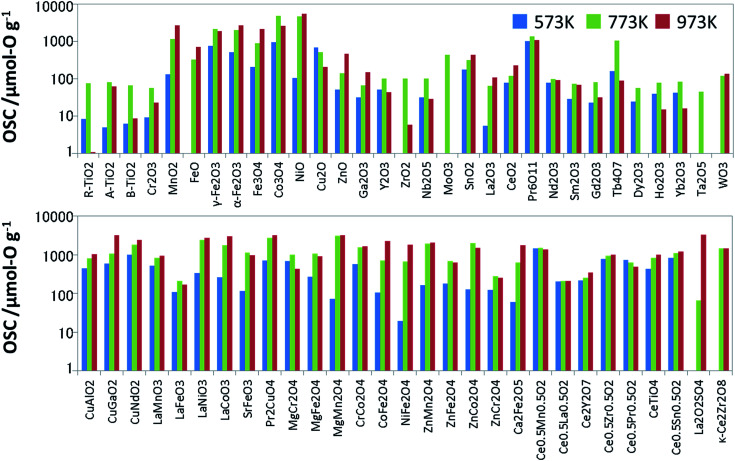
Measured OSC value of the Pd-loaded metal oxides used as the training data.

To prepare the training data for supervised learning, we measured not only known OSMs in the literature but also a wide range of oxides, and then synthesized 60 binary and ternary oxides using the solid phase reaction method or sol–gel method. All the metal oxides loaded with 1 wt% of Pd *via* impregnation. Since the dissociation of molecules is accelerated with the Pd support, it is expected to help an oxide material achieve its ideal OSC.^16^ The OSC was evaluated using a fixed-bed flow reactor. To burn off impurities on the surface, each oxide (2.0 g) loaded in the reactor was pretreated under a mixed gas stream of 5% oxygen and 95% nitrogen at a rate of 10 L min^−1^ at 300 °C for 5 min. The OSC was measured at 300 °C (573 K), 500 °C (773 K) and 700 °C (973 K). A cycle of 1% O_2_/N_2_ for 2 min and 2% CO/N_2_ for 2 min was repeated 6 times at each temperature at a flow rate of 10 L min^−1^. These experimental conditions were determined considering the fluctuation of the practical A/F ratio and previous papers.^17,18^ The amounts of CO_2_ emitted in circulating CO are averaged over the second to fifth cycles. The averaged value is divided by the weight of the sample, which is OSC [μmol-O g^−1^]. Generally speaking, OSC is classified into two categories: total OSC and dynamic OSC.^2,4^ The total OSC, which is in the equilibrium state, is the overall amount of stored oxygen not only on surface but also in bulk, while dynamic OSC is associated with the rate of oxygen release from OSMs and the mobility of oxygen. Under the above cycling conditions, we confirmed that OSC of conventional fluorite CeO_2_–ZrO_2_ reached equilibrium. Although it cannot promise for all the OSMs, it is considered that they have almost reached near equilibrium at worst. The measured OSC are summarized in Fig. 2 and Table S1 of ESI.† In particular, at 573 K, there were many materials which indicated OSC's below the measurement limit. The number of entries in the training data which could be used for the model training was 53 at 573 K, 60 at 773 K, and 57 at 973 K.

### Features

Next, the features that link OSC are defined. The reactions for storage and release of oxygen in the oxide can be expressed by the following chemical reaction formula.

1 MO_*x*−*δ*_ + *δ*/2O_2_ ↔ MO_*x*_

The enthalpy difference between the left and right states in eqn (1) corresponds to the formation energy of the oxygen defect. Therefore, the oxygen-defect formation energy is appropriate for an explanatory variable that accurately describes the characteristics of OSC. This quantity should be determined by the first-principles calculation of a large supercell made of repeatedly-connected unit cells to describe the isolated defects. This results in a high calculation cost. To accelerate the evaluation, Deml *et al.*^19^ performed the first-principles calculations on 45 oxide materials and used them as the training data for a linear regression model of the oxygen-defect formation energy, achieving an error range of 0.2 eV. Their model used three explanatory variables, which are: (i) heat of formation of the oxide, (ii) oxygen p-band center with the center of the band gap as the origin, and (iii) the average value of the electronegativity of the elements that constitute the polyhedron in the crystal structure. The features (i) and (ii) can be obtained by the first-principles calculation on a small unit cell, and (iii) can be easily obtained if the constituent elements and the crystal structure are known. Therefore, if the oxygen-defect formation energy can be predicted using (i) to (iii), the calculation cost is dramatically reduced in comparison to the direct calculation which uses the supercell.

It would be the best to be able to obtain the explanatory variables in a simple way. On the basis of the above insights, we examined the following seven explanatory variables of OSC:

*E_coh*: cohesive energy computed by Vienna *Ab initio* Simulation Package (VASP) code^20^ [eV]

*band gap*: Band gap (*E*_g_) [eV]

*p band center*: oxygen p-band center [eV] (with the top of the valence band as the origin)

*pband2*: (p band center) − (band gap)/2 [eV] (with center of the band gap (*E*_g_/2) as the origin)

*delta chi*: average value of the difference in electronegativity

*weight/O*: molecular weight per oxygen

*average r*: average distance between oxygen and the cation [Å]

Here we consider two cases of the definition of energy origins for the oxygen p-band center: the top of the valence band, and the middle of the band gap. Note that the *E_coh*, *band features* (*band gap* and p-band center or *pband2*), and *delta chi* correspond to the features of (i) to (iii) described above. Additionally, we added *weight/O* and *average r* as the features relating to the diversity of constituent elements and crystal structure.

To obtain these features, first-principles calculations were performed using VASP^20^ with PAW pseudo-potentials,^21^ which is based on the density functional theory. The Perdew–Burke–Ernzerhof (PBE)^22^ generalized-gradient-approximation functional was applied in the exchange and correlation energy terms. The cutoff energy of the plane-wave basis is 500 eV, with the k-point grid of the Brillouin zone divided into 0.01–0.02 Å^−1^. In the structural optimization, the atomic configurations were relaxed so that the force acting on each atom was 0.01 eV Å^−1^ or less. All the calculations were performed taking into consideration spin polarization.

### Machine learning model

**Fig. 3:**
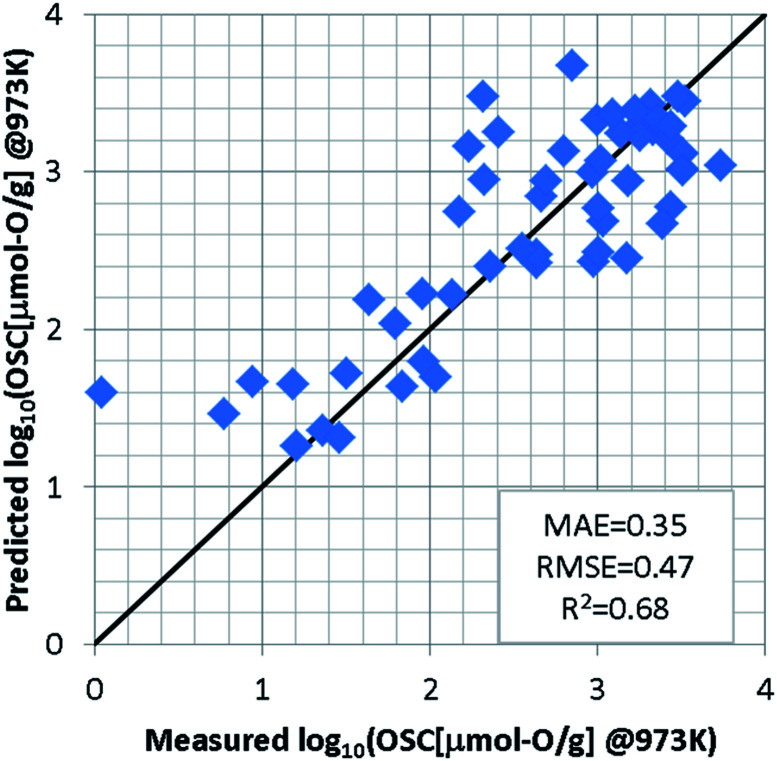
Results of leave-one-out cross validation by SVM regression model at 973 K.

For the regression model of OSC, we standardized the training dataset as mentioned in S2 of ESI† and used a support vector machine (SVM)^23,24^ which was implemented using the machine learning library scikit-learn.^25^ Since the number of detectably large OSC values depends on the measurement temperatures, an optimal model was constructed for each temperature. In each model, the explanatory variables were automatically selected by grid search so as to achieve the highest accuracy, as well as hyper-parameters of SVM. To evaluate the prediction accuracy, we used the leave-one-out cross validation (LOOCV). At 973 K for instance, the grid search selects the features *weight/O*, *E_coh*, and *pband2* as the appropriate explanatory variables. The SVM model was optimized with the Gaussian kernel by penalty term coefficient *C* = 1000, kernel parameter *γ* = 0.03, and width of insensitive loss function *ε* = 0.0001. Fig. 3 shows the results of LOOCV. The mean absolute error (MAE) and the root mean square error (RMSE) were 0.35 and 0.47, respectively. The results at 773 K and 573 K can be found in S3 of ESI.† To further validate this ML approach, the binary-classification task whether the OSC was more than a threshold value (we set it 900 μmol-O g^−1^ at 973 K) or not was also conducted as described in S4 of ESI.† Our model can screen the most likely high-capacity OSMs with more than 80% probability.

In addition to the SVM, we investigated the prediction accuracies using other machine learning (ML) algorithms; Gaussian Process Regression (GPR),^26^ Kernel Ridge Regression (KRR),^27^ Linear Ridge Regression (LRR),^28^ and Neural Network (NN).^29–32^ These algorithms were also implemented using the machine learning library scikit-learn.^25^ Table S5 in ESI† shows the prediction accuracy of LOOCV for various ML algorithms at each temperature. It is found that the prediction accuracy of SVM model is the best at each temperature. The radial basis function is used as the kernel in SVM, GPR, and KRR models. There is almost no difference in the prediction results of these three models. On the other hand, the prediction accuracy of LRR was worse. The poor prediction accuracy in NN model is due to the small training dataset. In general, the NN model requires a highly diverse training dataset with sufficient representative examples for proper prediction.^33^ In this study, the non-linear prediction model SVM seems to be appropriate.

Pairwise scatterplots for the OSC data at 973 K and seven explanatory variables are shown in Fig. S3 of ESI.† Here we discuss the details of the selected explanatory variables. Although the oxygen defect formation energy is considered to correlate with the OSC as mentioned in previous section, it is interesting that the “*E_coh*” and “*p band center*” have correlation with not only the oxygen defect formation energy but also the measured OSC. The correlation coefficients between the common logarithm of OSC (log_10_(OSC[μmol-O g^−1^]@973 K)) and these variables (*weight/O*, *E_coh*, and *pband2*) were −0.19, 0.45, and −0.10, respectively. Since OSC is standardized per oxide weight, it is reasonable that the *weight/O* feature is selected as the explanatory variable. The *E_coh* corresponds to the energy difference between the crystalline state and the isolated atoms. Therefore, the higher the *E_coh*, the more unstable the OSM will be. This result indicates that unstable OSMs would be preferable towards improving the OSC. The feature *pband2* is expressed by the oxygen p-band center and the band gap. The lower the oxygen p-band center, the more stable the oxygen states in the crystal. Since the correlation of OSC with this variable is negative, it indicates that oxygen should be stable within the crystal for higher OSC values. This result can be explained by the fact that moderate stability oxides are likely to release and store the oxygen molecules in reducing and oxidation atmospheres, respectively.

### Screening from database and experimental validation

**Fig. 4:**
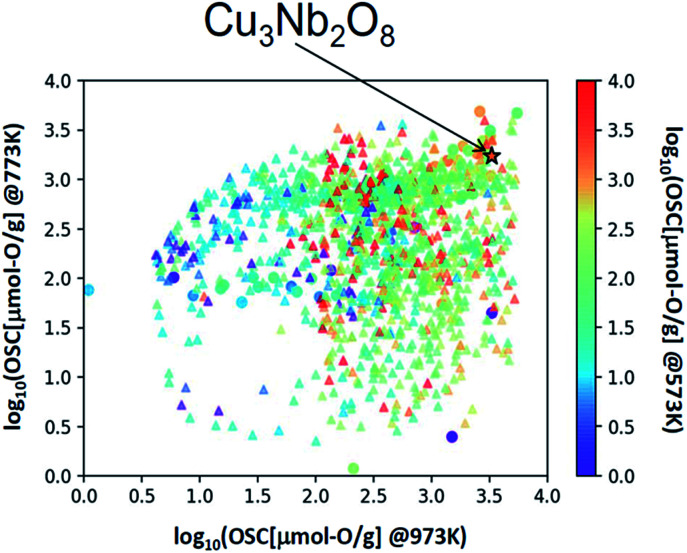
Scatter plot of OSC at 573, 773, and 973 K. △ is the predicted value for the material registered in the electronic structure calculation DB, ☆ is the predicted value of Cu_3_Nb_2_O_8_ and ○ represents the measured values of the training dataset.

We applied this regression model to the database (DB) of electronic-structure calculations of the 1300 oxides, in order to screen for high-capacity OSMs. Entries of this in-house DB were selected from the oxides which contained 100 or fewer atoms in their unit cell as registered in the inorganic crystal structure database (ICSD),^34^ owing to computational-cost restrictions. The scatter plot of the predicted OSC at each temperature is shown in Fig. 4. Among the candidate oxides, we focused on Cu_3_Nb_2_O_8_ because its predicted OSC was ranked relatively high at all temperatures and was comparatively easier to be synthesized. Whereas many of the OSMs reported so far contain scarce lanthanides (rare-earth elements) in CeO_2_–ZrO_2_ for example, Cu_3_Nb_2_O_8_ may offer an advantage because it is not composed of any lanthanides.

**Fig. 5:**
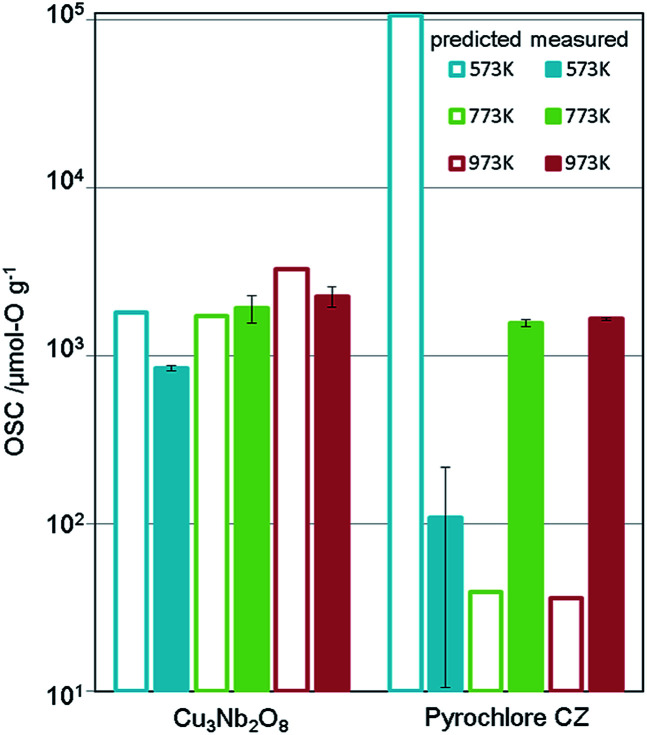
Predicted and measured values of OSC for Cu_3_Nb_2_O_8_ and pyrochlore-type CZ (the current material). Measured OSC is the mean value of two experimental results and error bars indicate the standard deviation.

The synthesis procedure is described in ESI.† X-ray diffraction (XRD) confirmed that a single phase was obtained. Fig. 5 shows the measurement results of the OSC along with those of a current high-performance OSM, pyrochlore-type CeO_2_–ZrO_2_ (p-CZ), for comparison. The present Cu_3_Nb_2_O_8_ showed a higher capacity than the baseline p-CZ at all temperatures. Furthermore, at 773 K, the predicted and measured values were in good agreement. On the other hand, the difference between the predicted and measured values of p-CZ is large, likely because the training dataset at 573 K includes only one pyrochlore-type crystal structure. The regression accuracy may improve with augmentation of the training dataset. Another reason seems that the regression model cannot learn the chemical insight of the redox of cations. For example, when the oxidation state of Ce in p-CZ changes from Ce^4+^ to Ce^3+^ during oxygen release, the theoretical maximum of OSC is 1693 [μmol-O g^−1^], while the prediction value at 573 K is higher than the theoretical maximum. Building the model considering the physical and chemical insights is one of the challenging problems in MI.

In addition, the rate of oxygen release/storage (OSC-r) was simply estimated from the transient response curve as shown in Fig. S4 of ESI.† The OSC-r for Cu_3_Nb_2_O_8_ and p-CZ were 2.38 × 10^−5^ mol min^−1^ and 1.60 × 10^−5^ mol min^−1^, respectively. Surprisingly, Cu_3_Nb_2_O_8_ has better performance than p-CZ from the view point of rate, though the OSC-r is not predicted in our ML model directly. Regarding the OSC-r estimated from Fig. S4 of ESI,† the OSC-r of p-CZ is increasing monotonically as the temperature increases from 573 K to 973 K. On the other hand, the OSC-r of Cu_3_Nb_2_O_8_ at 773 K is highest among three temperatures. This performance of Cu_3_Nb_2_O_8_ is considered to be related to the possibility of phase separation and thermal stability. Nonetheless, the industrial use of an OSM requires other performance metrics such as thermal stability and easy synthesis by mass production, which are not considered in this virtual screening.

**Fig. 6:**
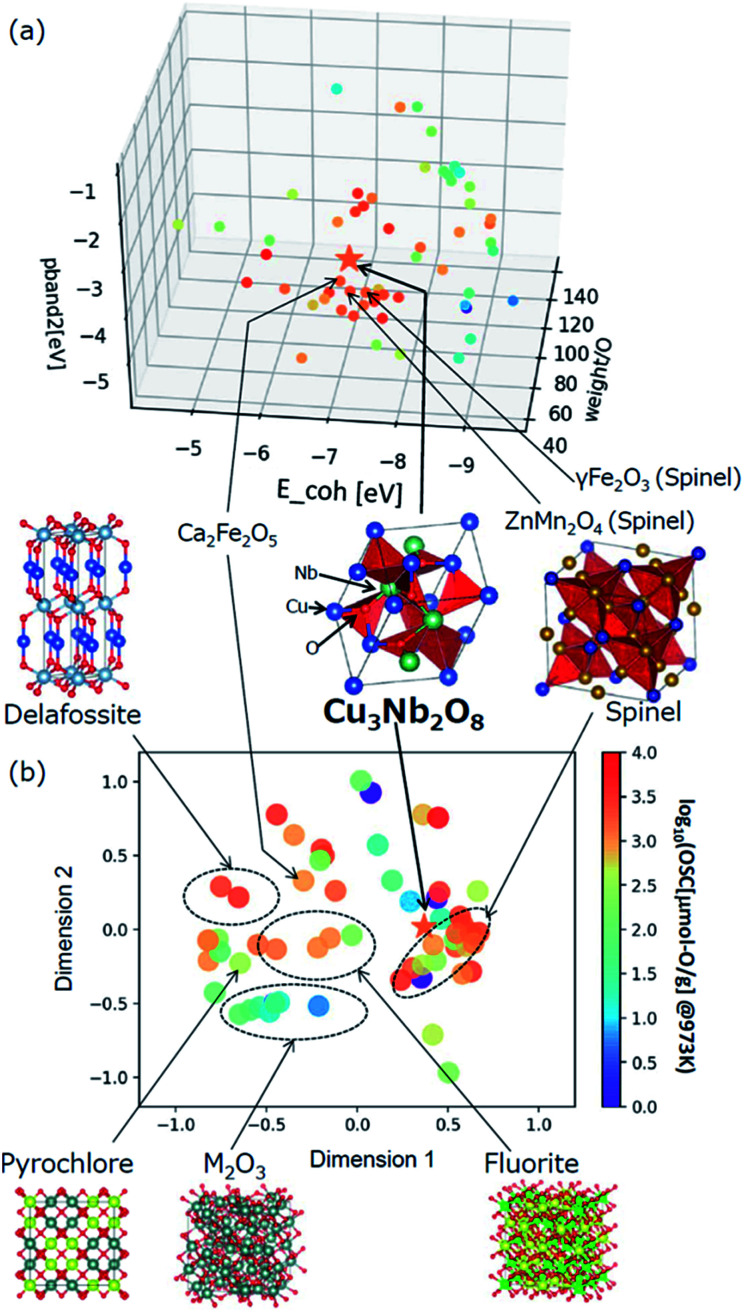
(a) Scatter plot of the selected three explanatory variables, *weight/O*, *E_coh*, and *pband2* for the training data and Cu_3_Nb_2_O_8_. (b) Multidimensional scaling plot of the SOAP distance between the training dataset and Cu_3_Nb_2_O_8_ based on the similarity of crystal structure.

The selected explanatory variables and crystallographic features of Cu_3_Nb_2_O_8_ were compared to the training data in Fig. 6. The color of each point in Fig. 6 represents the measured value of OSC[μmol-O g^−1^] at 973 K. The 3-dimentional scatter plot in the features space (Fig. 6(a)) indicates that Cu_3_Nb_2_O_8_ is the most similar to Ca_2_Fe_2_O_5_ among training datasets. The second-most similar materials are γ-Fe_2_O_3_ and ZnMn_2_O_4_ which are classified in spinel-type structure. This result indicates that the Cu_3_Nb_2_O_8_ is similar to the spinel-type OSMs in terms of not only crystal structure (to be mentioned later) but also the material features space. In addition, we applied the Smooth Overlap of Atomic Positions (SOAP) kernel,^35,36^ which describes the similarities of the local structures surrounding oxygen atoms. The structural similarity between the 60 oxides belonging to the training dataset and Cu_3_Nb_2_O_8_ was calculated as a form of SOAP distance matrix. Parameters of the SOAP kernel, the cutoff radius and width of Gaussian distribution of atoms, were set at 5 Å and 0.5 Å, respectively. To distinguish the elemental species in the SOAP measure, we include the electronegativity in the width of Gaussian function at 1, as in the ref. 36. Fig. 6(b) shows a reduced two-dimensional map of the SOAP distance matrix projected using the metric multi-dimensional scaling method^37^ implemented in the scikit-learn library. The training dataset can be classified in terms of their crystal structures: *e.g.* fluorite (such as CeO_2_), delafossite, and spinel types. Cations around the oxygen ion in spinel oxides form edge and vertex sharing tetrahedral sites. The discovered Cu_3_Nb_2_O_8_, which is not denoted by the spinel, has a similar structure but consists of the tetrahedral and triangular sites. This similarity is detected in the SOAP distances. This result suggests that utilizing MI highlights hidden OSMs that are structurally similar to the known materials, but difficult for humans to recognize only with the names of the structure types. There may still exist prospective hidden OSMs whose crystal structures have received little attention so far.

## Conclusions

In conclusion, we have demonstrated a data-driven material search using the experimental OSC data prepared under highly controlled uniform conditions. This MI scheme can significantly accelerate the identification and development of new OSMs. Indeed, by screening the existing oxides, we discovered a novel high-capacity OSM, Cu_3_Nb_2_O_8_.

The oxygen storage and release reaction mechanism of the proposed material Cu_3_Nb_2_O_8_ is currently under investigation. In particular, the oxidation state change of Cu accompanying the generation of oxygen defects is important for the industrial use. Detailed reaction mechanisms, phase separation, and thermal stability of Cu_3_Nb_2_O_8_ will be reported in the future.

Furthermore, experimental validation of high OSC candidates other than Cu_3_Nb_2_O_8_ is also underway. Augmentation the training data with these validated ones makes virtual screening process a closed-loop. This is expected to improve the accuracy of the prediction model and further advance the material discovery.

## Conflicts of interest

There are no conflicts to declare.

## Supplementary Material

RA-009-C9RA09886K-s001
