# Supplementary material for: Search for high-capacity oxygen storage materials by materials informatics
Source: RSC Adv. 2019 Dec 17;9(71):41811–6. doi: 10.1039/c9ra09886k (PMC9076568; doi:10.1039/c9ra09886k)
Supplement: RA-009-C9RA09886K-s001 [file RA-009-C9RA09886K-s001.pdf]

Electronic Supplementary Material (ESI) for RSC Advances.
This journal is © The Royal Society of Chemistry 2019

Supporting Information

Search for High-capacity Oxygen Storage Materials by Materials Informatics

Nobuko Ohba,[*a] Takuro Yokoya,[b] Seiji Kajita,[a] and Kensuke Takechi[a]*

[a] Toyota Central R&D Labs., Inc., Nagakute, Aichi 480-1192, Japan

[b] Toyota Motor Corporation Higashi-Fuji Technical Center, Susono, Shizuoka 410-1193, Japan

S1. Training dataset

Table S1. Measured OSC value of the Pd-loaded metal oxides used as the training data. The horizontal bars — indicate OSC's below the measurement limit.

material	OSC@573K /μmol-O g^{-1}	OSC@773K /μmol-O g^{-1}	OSC@973K /μmol-O g^{-1}	material	OSC@573K /μmol-O g^{-1}	OSC@773K /μmol-O g^{-1}	OSC@973K /μmol-O g^{-1}
R-TiO_2	8.45	75.8	1.1	CuAlO2	449	816	1040
A-TiO_2	4.94	82.3	61.7	CuGaO2	599	1080	3210
B-TiO_2	6.22	66.6	8.78	CuNdO2	1000	1840	2460
Cr_2O_3	9.37	57	23.1	LaMnO3	533	849	948
MnO_2	133	1168	2716	LaFeO3	111	211	172
FeO	—	325	711	LaNiO3	339	2440	2750
γ-Fe_2O_3	757	2167	1866	LaCoO3	266	1800	3020
α-Fe_2O_3	510	2031	2647	SrFeO3	116	1160	996
Fe_3O_4	209	878	2138	CuPr2O4	720	2730	3210
Co_3O_4	939	4867	2606	MgCr2O4	686	1020	434
NiO	105	4691	5465	MgFe2O4	273	1070	932
Cu_2O	695	511	208	MgMn2O4	72.6	3110	3200
ZnO	51.7	139	461	CrCo2O4	573	1570	1680
Ga2O3	31.9	67	151	CoFe2O4	106	712	2270
Y2O3	51.4	101	43.5	NiFe2O4	19.8	674	1820
ZrO_2	—	102	5.97	ZnMn2O4	164	1950	2080
Nb_2O_5	31.7	102	28.9	ZnFe2O4	182	695	627
MoO_3	—	432	—	ZnCo2O4	127	1990	1530
SnO_2	175	313	435	ZnCr2O4	126	280	257
La_2O_3	5.54	64.6	108	Ca2Fe2O5	61.4	636	1780
CeO_2	78	119	229	Ce0.5Mn0.5O2	1470	1540	1370
Pr_6O_{11}	997.8	1344.8	1072	Ce0.5La0.5O2	207	210	212
Nd_2O_3	78.9	98.9	92.2	Ce2Y2O7	219	255	355
Sm_2O_3	28.4	73	69	Ce0.5Zr0.5O2	802	946	1010
Gd_2O_3	23	81.4	32	Ce0.5Pr0.5O2	748	626	490
Tb_4O_7	159	1037	89.9	CeTiO4	436	854	1010
Dy_2O_3	24.8	55.99	—	Ce0.5Sn0.5O2	829	1110	1230
Ho_2O_3	39.7	78.8	15.2	La2O2SO4	—	67.3	3310
Yb_2O_3	42.7	84.8	16.1	κ-Ce2Zr2O8	—	1500	1500
Ta_2O_5	—	45	—				
WO_3	—	120	135				

S2. Data scaling

Since the measured data of OSC distributed in a range of 5 to 6000 μmol/g, logarithms were applied to standardize them. Additionally, for each explanatory variable (x), another standardization was performed using the mean value μ and the variance σ of the explanatory variables of the training data, as

$$\bar{x} = (x - \mu)/\sigma \ . \qquad \text{(S1)}$$

In the case of the database (DB), which is screened by the trained model, the explanatory variables may spread to a larger range than that of the training data, even using the standardizing of Eq. (S1), because the DB records a large number of materials. In other words, this issue implies that the majority of the DB entries would lie outside of the range that the prediction model was trained on. To minimize this extrapolation problem, we applied variable transformation as $\tilde{x} = \tanh \bar{x}$ to restrict the range of standardized features between -1 and +1.

S3. Results of leave-one-out cross validation

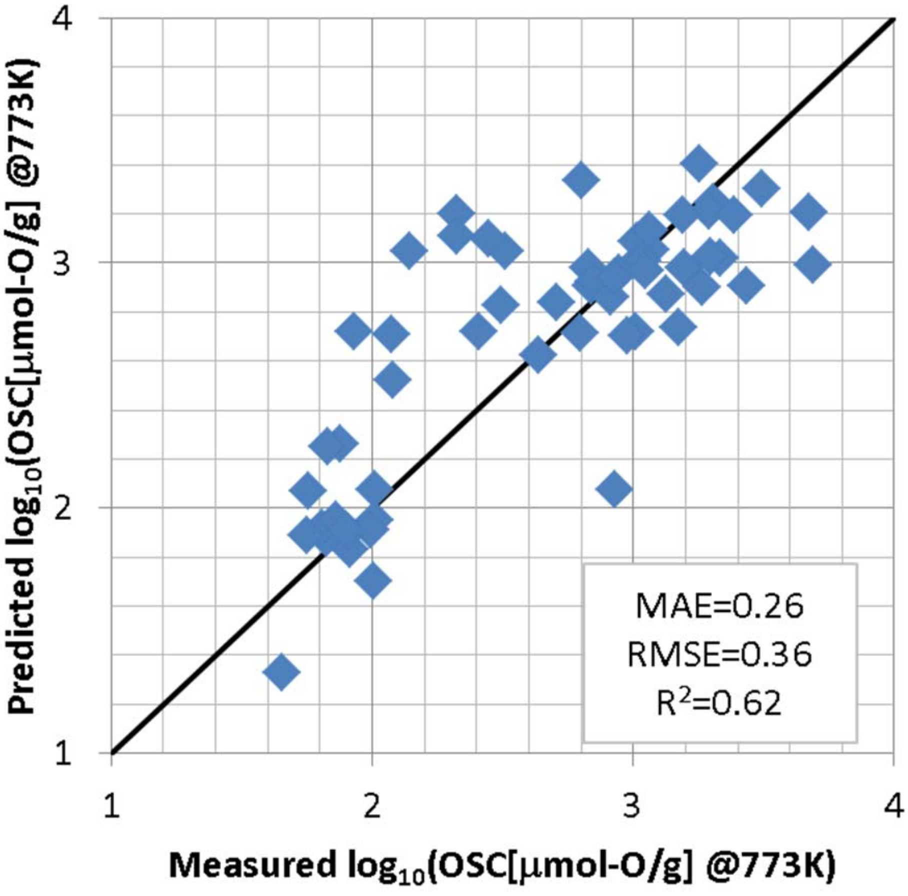

Figure S1. Results of leave-one-out cross validation by SVM regression model at 773 K

Table S2. The extracted features and hyperparameters of SVM regression model at 773 K

Extracted features	Hyperparameter	
• average r<br>• E_coh<br>• pband center<br>• Band gap [eV]	kernel	rbf
	C	100
	γ	0.05
	ε	0.00001

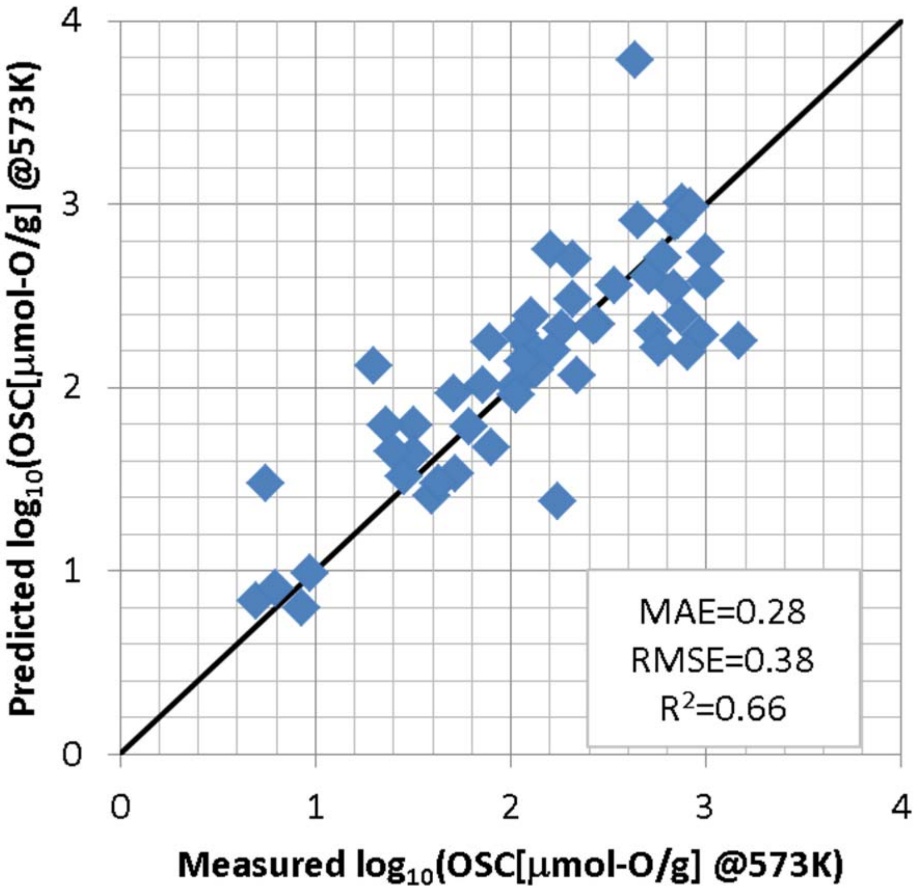

Figure S2. Results of leave-one-out cross validation by SVM regression model at 573 K

Table S3. The extracted features and hyperparameters of SVM regression model at 573 K

Extracted features	Hyperparameter	
• E_coh<br>• delta chi<br>• pband2	kernel	rbf
	C	1000
	γ	0.3
	ε	0.0001

S4. Binary-classification task

Table S3 shows the accuracy and F-score of LOOCV for the binary-classification task at each temperature. Threshold values (OSCth) used to divide the measured OSC for 60 oxides into two labeled classes are also shown in Table S4. The SVM classification models were built with selected features in the regression model at each temperature. The number of training data values greater than OSCth at 973K, 773K, and 573K are 29, 30, and 27, respectively.

Table S4. The accuracy and F-score of binary-classification task

Temperature	OSCth (μmol-O/g)	Accuracy	F-score
973K	900	0.81	0.81
773K	650	0.83	0.84
573K	150	0.79	0.79

S5. Accuracy for machine learning algorithm

Table S5 shows the prediction accuracy of LOOCV for various machine learning (ML) algorithms at each temperature. We chose Support Vector Machine (SVM), Gaussian Process Regression (GPR), Kernel Ridge Regression (KRR), Linear Ridge Regression (LRR), and Neural Network (NN) as ML algorithms. The prediction accuracy of SVM model is the best among five ones at each temperature. The radial basis function is used as the kernel in SVM, GPR, and KRR models. There is almost no difference in the prediction results of these three models.

Table S5. Prediction results for various machine learning algorithms. Unit of mean absolute error (MAE) and root mean squared error (RMSE) of the regression is $\log_{10}$(OSC[μmol-O/g]).

Temperature	ML algorithm	MAE	RMSE	R^2
973K	SVM	**0.35**	**0.47**	**0.68**
	GPR	0.40	0.52	0.60
	KRR	0.39	0.51	0.61
	LRR	0.54	0.69	0.31
	NN	0.61	0.77	0.13
773K	SVM	**0.26**	**0.36**	**0.62**
	GPR	0.28	0.37	0.61
	KRR	0.30	0.39	0.59
	LRR	0.35	0.44	0.45
	NN	0.40	0.49	0.30
573K	SVM	**0.28**	**0.38**	**0.66**
	GPR	0.38	0.48	0.45
	KRR	0.38	0.48	0.46
	LRR	0.48	0.49	0.19
	NN	0.55	0.69	0.03

SVM : Support Vector Machine, GPR : Gaussian Process Regression,
KRR : Kernel Ridge Regression, LRR : Linear Ridge Regression, NN : Neural Network.

S6. Pairwise scatterplots

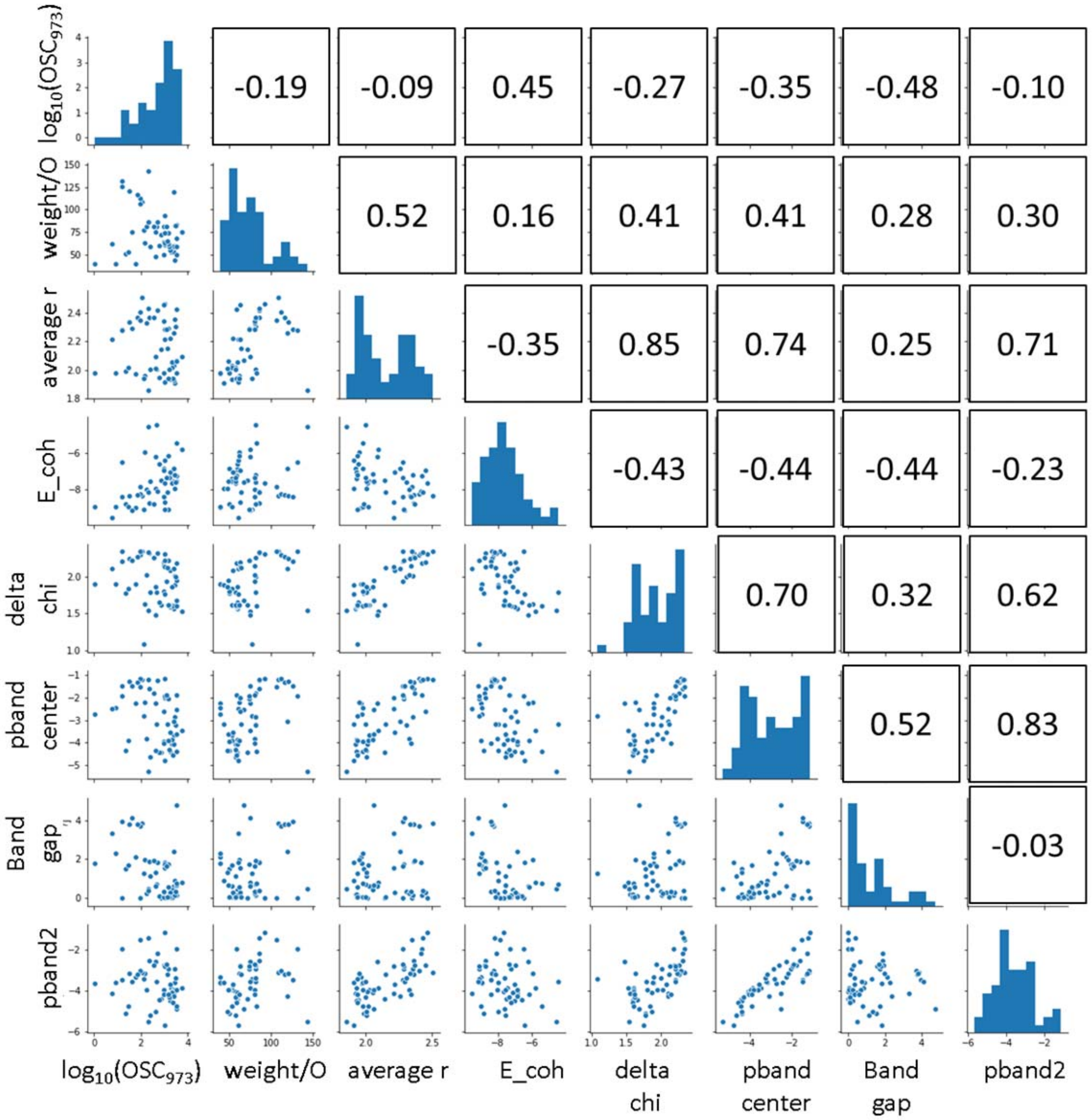

Figure S3. Pairwise scatterplots for the OSC data at 973K and seven explanatory variables

S7. Rate of OSC

Figure S4 shows the transient response curve for the concentrations of CO, CO_2, and O_2 for $Cu_3Nb_2O_8$ and p-CZ. We simply estimated the rate of OSC (OSC-r) by the slope of the starting point of the CO_2 generation in Fig. S4. The estimated OSC-r for p-CZ and $Cu_3Nb_2O_8$ were 1.60×10^{-5}mol/min and 2.38×10^{-5}mol/min, respectively. Therefore, proposed $Cu_3Nb_2O_8$ has better performance than p-CZ from the view point of rate.

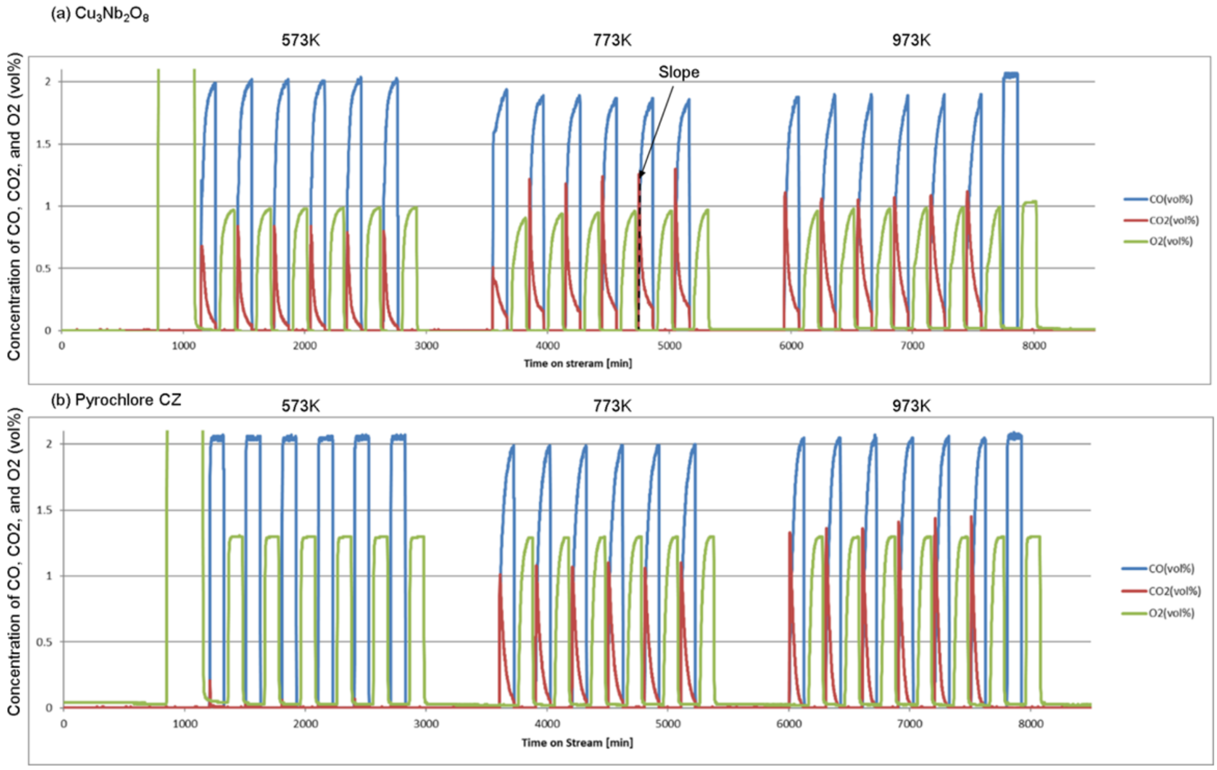

Figure S4. The transient response curve for the concentrations of CO, CO_2, and O_2 during a cycle of 1% O_2/N_2 for 2min and 2% CO/N_2 for 2min

S8. Synthesis of Pd(1wt%)/Cu3Nb2O8

We synthesized $Cu_3Nb_2O_8$ using the solid phase reaction method. Binary oxides (Cu_2O and Nb_2O_5), used as the raw material, were ground and mixed for 1 h at a mole ratio of Cu_2O/Nb_2O_5 of 1.5 using an agate mortar. The material was pressed at 196 kN using the cold isostatic pressing method and pelletized. The pellet was air-fired at 800℃ for 36 h and subsequently air-fired at 950℃ for 40 h to obtain the oxide. The resulting pellet-shaped oxide was ground in an agate mortar and loaded with 1wt% of Pd via impregnation method. This oxide was suspended in a little water and a nitric acid solution of palladium nitrate was dripped on it. The suspension was subsequently heated at 120℃ to vaporize the water. The Pd(1wt%)/ $Cu_3Nb_2O_8$ was obtained after being reduced by hydrogen at 400 °C in air for 2h.
